# Building Reflective Practice: Establishing a Sustainable Balint Group Model for Child and Adolescent Psychiatrists in Wales

**DOI:** 10.1192/bjo.2025.10266

**Published:** 2025-06-20

**Authors:** Megan Davies-Kabir, Andrew Todd

**Affiliations:** Aneurin Bevan University Health Board, Caerleon, United Kingdom

## Abstract

**Aims:** The project aimed to establish a regular Balint group for Child and Adolescent Psychiatry (CAP) Specialist Resident (SpR) doctors in Wales, support the development of future Balint leaders through training, and enhance the sustainability of these groups. By increasing the number of accredited leaders across Wales, the project sought to expand opportunities for clinicians at various levels to engage in reflective practice, have a safe space to consider their relationship with patients, and ultimately increase levels of emotional capacity and wellbeing.

**Methods:** In July 2023, following notification of funding, interest was canvassed among CAP SpRs in Wales, resulting in the formation of a group of five SpRs committed to becoming accredited Balint leaders. Regular online meetings were set up for mutual support and to facilitate progress towards UK Balint Society accreditation. An online fortnightly term-time Balint group was organized for CAP psychiatrists, with an accredited leader overseeing the sessions. Despite initial challenges accessing funds, the project progressed, and the Balint group commenced in November 2023. Two members attended a Balint training day in December 2023, despite financial constraints, while plans to use funds for further events and supervision encountered delay due to complex bureaucratic pathways.

**Results:** A total of 16 child psychiatrists from Wales participated in the Balint group, with 12 (mostly SpRs) attending regularly. Feedback from 11 participants indicated overwhelmingly positive responses to the group’s impact on their practice and well-being, with the majority expressing that it enhanced their understanding of the doctor-patient relationship and provided a safe space for emotional reflection. Key strengths identified included the supportive environment and the opportunity for group reflection. While online participation worked well for most, there were suggestions for occasional face-to-face meetings. Notably, two of the initial Balint leadership cohort are now leading the group following the funding phase, demonstrating the project’s potential for sustainability.

**Conclusion:** The project successfully introduced Balint group practice to CAP SpRs in Wales and initiated a pathway for future leaders. Despite challenges with funding and administrative processes, the initiative has had a positive impact on SpRs’ professional development, wellbeing, and reflective practice. The creation of a sustainable model for leadership development is a significant outcome. The long-term success will depend on securing ongoing funding for training and supervision. Increased numbers of accredited leaders are crucial for expanding access to these valuable groups. Ongoing efforts to address financial barriers are necessary for sustaining and expanding the initiative.

